# Complex transcription regulation of acidic chitinase suggests fine-tuning of digestive processes in *Drosera binata*

**DOI:** 10.1007/s00425-025-04607-2

**Published:** 2025-01-12

**Authors:** Veronika Mikitova, Martin Jopcik, Miroslav Rajninec, Jana Libantova

**Affiliations:** Institute of Plant Genetics and Biotechnology, Plant Science and Biodiversity Center, Slovak Academy of Sciences, Akademicka 2, P. O. Box 39A, 950 07 Nitra, Slovak Republic

**Keywords:** ß-1,3-Glucan, Carnivorous plants, Chitin, Enzymatic parameters, Glucose, Pachyman

## Abstract

**Main conclusion:**

DbChitI-3, *Drosera binata*'s acidic chitinase, peaks at pH 2.5 from 15 °C to 30 °C. Gene expression is stimulated by polysaccharides and suppressed by monosaccharide digestion, implying a feedback loop in its transcriptional regulation.

**Abstract:**

Here, we characterised a novel chitinase gene (*DbChitI-3*) isolated from the carnivorous plant species *Drosera binata* with strong homology to other *Drosera* species' extracellular class I chitinases with a role in digestive processes. The capability to cleave different forms of chitin was tested using recombinantly produced chitinase in *Escherichia coli* (rDbChitI-3^S−^His) and subsequent purification. The recombinant protein did not cleave chitin powder, the mono-, di- and tri- N-acetyl-D-glucosamine substrates, but cleaved acetic acid-swollen chitin. Fluorometric assay with acetic acid-swollen FITC-chitin as a substrate revealed the maximum enzyme activity at pH 2.5, spanning from 15 °C to 30 °C. Comparing enzymatic parameters with commercial chitinase from *Streptomyces griseus* showed rDbChitI-3S-His efficiency reaching 64.3% of *S. griseus* chitinase under optimal conditions. The highest basal expression of *DbChitI-3* was detected in leaf blades. In other organs, the expression was either fivefold lower (petioles) or almost nondetectable (stems, roots and flowers). Application of gelatin, chitin, and pachyman resulted in a 3.9-, 4.6- and 5.7-fold increase in the mRNA transcript abundance of *DbChitI-3* in leaves. In contrast, monosaccharides and laminarin decreased transcription of the *DbChitI-3* gene by at least 70%, 5 h after treatment. The simultaneous application of suppressor and inducer (glucose and pachyman) indicated the predominant effect of the suppressor, implying that sufficient monosaccharide nutrients prioritize absorption processes in *D. binata* leaves over further digestion of the potential substrate.

**Supplementary Information:**

The online version contains supplementary material available at 10.1007/s00425-025-04607-2.

## Introduction

*Drosera* L. (sundew) is one of the largest genera of carnivorous plants with more than 250 tropical and temperate species (Gonella et al. 2015). *Drosera* spp. occur primarily in swamps and bogs, a moist, sunny habitat with acidic, nutrient-poor soils (Givnish et al. 1984). All members of the genus *Drosera* form specialized structures called “flytraps”, which are formed from glandular trichomes developed in the leaf epidermis (Baek and Kim 2008; Ellison and Gotelli 2009).

The new leaves sprout from a very short stem. Initially, they emerge as tightly coiled structures, gradually unrolling as they develop. Each matured leaf has a cylindrical petiole that splits into two, giving a forked appearance. The flattened blades of the fork are covered from the axis to the tip with fine, red, mucilage-tipped hairs, tentacles. These become very active in contact with prey and stimulate the rapid movement of other tentacles towards the captured insects. The leaf then curls in on itself and envelops the prey for digestion (Gilbert 1984). The mucilage of carnivorous plants is highly viscous, very sticky, and contains a cocktail of hydrolytic enzymes that decompose captured prey (Juniper et al. 1989; Adlassnig et al. 2011). Some of the genes for digestive enzymes, such as chitinases, have been shown to have evolved from genes responsible for defence against parasites and invading pathogens (Schulze et al. 2012; Wheeler and Carstens 2018). They degrade the chitin-rich exoskeleton of arthropods, allowing proteases, β−1,3-glucanases, lipases, and nucleases to penetrate and break down internal tissues (Paszota et al. 2014).

Plant chitinases belong to the glycoside hydrolases (GH) family 18 and 19, further classified into seven classes. Chitinases classes I, II, IV and VI consist of the GH19 catalytic domain and the N-terminal chitin-binding domain, which supports hydrolysis of the substrate (Ohnuma et al. 2012). The chitinases of classes II and VII have a GH19 catalytic domain exclusively, and classes III and V have a GH18 catalytic domain. The individual classes of chitinases differ in their expression profile (Kesari et al. 2015), enzyme activity and substrate specificity (Unhelkar et al. 2017). In non-carnivorous plants, most of them are involved in defence processes against pathogens, especially in response to fungal attack (van Loon et al. 2006; Schuhegger et al. 2006), but chitin-degrading enzymes have also been found in the digestive fluids of carnivorous plants (Eilenberg et al. 2006; Hatano and Hamada 2008; Renner and Specht 2012; Paszota et al. 2014; Filyushin et al. 2019; Sinelnikov et al. 2020).

Recently, we isolated the gene for a class I extracellular chitinase (*DrChit*) from the carnivorous *Drosera rotundifolia* plant and defined its expression profile. The gene was active almost exclusively in leaves, while a non-specific inducer (sand and gelatin) increased its expression two-fold and a specific substrate, chitin, seven-fold (Jopcik et al. 2017). Corresponding purified chitinase protein (DrChit) effectively degraded chitin powder and exhibited maximum activity at pH 6.0.

In this study, we aimed to characterize the gene for chitinase class I (*DbChitI-3*) from the carnivorous plant *D. binata*. By comparing the expression profiles and regulatory mechanisms of *DbChitI-3* and *DrChit*, we sought to uncover functional similarities and differences between *D. binata*, native to Australia and nearby islands, and *D. rotundifolia*, which is distributed across the northern hemisphere. Additionally, we investigated the effects of various potential inducers and suppressors on *DbChitI-3* gene expression to identify novel regulatory mechanisms governing hydrolase genes involved in prey digestion. Analysis of purified recombinant DbChitI-3 protein revealed its enzymatic activity under highly acidic conditions and its distinct substrate specificity compared to other *Drosera* species. These findings provide valuable insights into the evolutionary adaptations of carnivorous plants for nutrient acquisition and may have broader implications for biotechnology, such as the development of specialized enzymes for industrial applications or enhanced agricultural pest control strategies.

## Materials and methods

### Plant material

Plants of *Drosera binata* were a gift from the Institute of Experimental Botany of the Czech Academy of Sciences and cultivated in vitro on ¼ Murashige & Skoog medium (Duchefa), supplemented with Gamborg B5 vitamin mixture (Duchefa), 2% (w/v) sucrose and 0.7% (w/v) agar pH 5.7 at 20 ± 2 °C, with a 16 h photoperiod and a light level of 50 µmol m^−2^s^−1^. Leaf blades, petioles, roots and flowers of non-treated and treated in vitro-grown plants were used to analyze expression profiles. For the treatment, chitin from crustacean shells (Sigma-Aldrich), cellulose powder (Merck), gelatin (Serva), sand (Sigma-Aldrich), laminarin (Sigma-Aldrich), pachyman (Megazyme) and the mixture pachyman (Megazyme): chitin (Sigma-Aldrich) (1:1, w/w) (in solid form) and the mixture 100 mM glucose: pachyman (in solid form) were applied to the leaves for 24 h. Pachyman was also tested as an inducer of chitinase gene transcription for 5 h, 24 h and 48 h. The solution of 2% (w/v) laminarin (Sigma-Aldrich), 100 mM D( +)-glucose (Merck), and 100 mM N-acetyl-D-glucosamine (Sigma-Aldrich) were applied to leaves for 1 h, 5 h and 24 h. In addition, 10 mM D( +)-glucose (Merck) was tested for 24 h. The control and treated leaves samples were then frozen in liquid nitrogen and stored at – 80 °C.

### Isolation of a genomic clone of *DbChitI-3*

The genomic DNA of *D. binata* was isolated from the leaves (1 g), according to Bekesiova et al. (1999). The internal fragment of the *DbChitI-3* gene was amplified in a 50 µL solution containing 100 ng DNA template, 15 pmol P1–P2 primers (Table S1), 400 µM dNTP, 1 × Synergy reaction buffer, and 1U SynergyT DNA polymerase (GeneCraft). The first step at 95 °C for 3 min was followed by 40 cycles at 94 °C for 30 s; 55 °C for 30 s; 72 °C for 1 min; and a final extension at 72 °C for 10 min. The PCR product was cloned into pJET1.2 vector (Thermo Fischer Scientific) and commercially sequenced.

We used the Genome Walker kit to determine the full-length continuous sequence (Clontech). To obtain the 5' upstream sequence, genomic DNA was digested by *Eco*RV. Adaptor DNA provided in the kit was ligated to *Eco*RV–digested genomic DNA fragments. The used primers are listed in Table S1. The first-round PCR was conducted using an adaptor primer AP1FOR provided with the kit and a gene-specific P3REV primer. The second-round PCR was performed using the nested adaptor AP2FOR primer and the P4REV primer. The isolated PCR fragment with an unknown sequence at the 5' end was sequenced and used to design the gene-specific primers P5REV and P6REV. To extend the 5' upstream sequence containing the 5' regulatory sequence, Genome Walking-PCR was performed on the *Dra*I library using the same forward adaptor primers and P5REV and P6REV primers in the first- and second-round PCR, respectively. Further extension of the 5' upstream sequence was performed on the *Stu*I library using the same forward adaptor primers and P7REV and P8REV primers in the first- and second-round PCR, respectively. To obtain the 3' downstream DNA sequence, the genomic DNA was digested with *Eco*RV. The first- and second-round PCRs were conducted using the P9FOR–AP1REV and P10FOR–AP2REV primers, respectively. The PCR conditions were one cycle at 94 °C for 2 min; followed by seven cycles at 94 °C for 25 s; 72 °C for 3 min; 32 cycles at 94 °C for 25 s, 67 °C for 3 min, and a final extension at 67 °C for 7 min. The DNA amplified by PCR was cloned into pGEM-T Easy vector (Promega) and sequenced.

The complete genomic *DbChitI-3* sequence was amplified on genomic DNA using the P11FOR–P12REV primers and the following conditions: 94 °C for 3 min followed by 35 cycles at 94 °C for 30 s, 65 °C for 40 s and 72 °C for 3 min. The final elongation was performed at 72 °C for 10 min.

### Isolation of *DbChitI-3* cDNA clone

Total RNA was extracted from the leaves of in vitro cultivated *D. binata* plants using the protocol described by Bekesiova et al. (1999). Genomic DNA was removed from RNA with RNase-free DNaseI (Thermo Fischer Scientific). The quality and quantity of the extracted RNA was checked spectrophotometrically using DeNovix DS-11.

To obtain the full-length cDNA of the *DbChitI-3* gene, 3'- and 5'- cDNA ends were isolated using a GeneRacer kit (Invitrogen), according to the manufacturer's instructions. First-strand cDNA was synthesized on five µg of total RNA using the GeneRacer oligo dT primer. 5´-RACE (rapid amplification of cDNA end) was performed in two successive PCRs with P13FOR–P14REV and P15FOR–P16REV (Table S1), respectively. Similarly, 3´-RACE was performed in two subsequent PCRs with P17FOR–P18REV and P19FOR–P20REV primers, respectively (Table S1). The PCR involved one cycle at 94 °C for 2 min, followed by 35 cycles of 94 °C for 30 s, 60 °C for 30 s, 72 °C for 1 min, and a final elongation step at 72 °C for 7 min. PCR products of the expected sizes were extracted from the gel, inserted into a cloning vector, and commercially sequenced.

The chitinase coding sequence (*cdsDbChitI-3*) was amplified using the P21FOR–P22REV primers on the cDNA template at the conditions of 94 °C for 3 min, followed by 45 cycles of 94 °C for 30 s, 60 °C for 30 s and 72 °C for 90 s. The last step was carried out at 72 °C for 10 min. Next, the PCR product was cloned into a pJET 1.2 vector (Thermo Fischer Scientific) and sequenced.

### Bioinformatic analysis of sequence data

Sequence alignments were performed using the CLUSTALW software (Thompson et al. 1994). Similarity searches for nucleotide and amino acid sequences of DbChitI-3 were carried out using the BLASTn, BLASTx and BLASTp programs (http://www.ncbi.nlm.nih.gov/BLAST/) (Altschul et al. 1990). The intron–exon structure of the *DbChitI-3* gene was analyzed using NetGene2 (http://www.cbs.dtu.dk/services/NetGene2/ (Hebsgaard et al. 1996). The nucleotide sequence of the *cdsDbChitI-3* was translated into amino acid sequence using the ExPASy Translate Tool (http://web.expasy.org/translate/) (Gasteiger et al. 2003). Motif analysis was conducted using the InterProScan software (http://www.ebi.ac.uk/InterProScan) (Quevillon et al. 2005) (Table S2). Prediction of the signal peptide's presence and cleavage site was analyzed using the SIGNALP 4.1 program (http://www.cbs.dtu.dk/services/SignalP/) (Petersen et al. 2011). The deduced amino acid sequence was assessed for possible glycosylation and phosphorylation sites using GlycoEP (http://www.imtech.res.in/raghava/glycoep/) (Chauhan et al. 2013) and NetPhos 2.0 (http://www.cbs.dtu.dk/services/NetPhos/) (Blom et al. 1999). Subcellular localization of the protein was predicted using the TARGETP 1.1 (http://www.cbs.dtu.dk/services/TargetP/) (Emanuelsson et al. 2007) and Psort (http://psort.hgc.jp/form.html) (Horton et al. 2007) programs. The eukaryotic promoter and transcription initiation site were predicted using the Neural Network Promoter Prediction (http://www.fruitfly.org/seqtools/promoter) (Reese 2001). In silico analysis of the 5'- and 3'-untranslated regions were conducted using the software PLACE (http://www.dna.affrc.go.jp/ PLACE /signalscan.html/) (Higo et al. 1999). For phylogenetic analysis, sequences were aligned using the MUSCLE algorithm (Edgar 2004) with default parameters and the Neighbour-joining tree was constructed with MEGA X software (http://www.megasoftware.net) (Kumar et al. 2018). The bootstrap consensus tree inferred from 1000 replicates (Felsenstein 1985) was taken to represent the evolutionary history of the taxa analyzed.

### Recombinant production of the rDbChitI-3^S−^His in *E. coli* and its purification

The open reading frame of the *D. binata* chitinase gene without the signal peptide** (DbChitI-3**^*−S*^) was amplified by PCR using the primers P27FOR–P28REV (Table S1), both with *Pml*I restriction sites on the template of cDNA *DbChitI-3*. The PCR program ran at 95 °C for 3 min, followed by 32 cycles at 95 °C for 30 s, 62 °C for 30 s, and 72 °C for 1 min. The final extension was performed at 72 °C for 10 min. The PCR product was digested with *Pml*I and ligated into a pET-K2 bacterial expression vector, a derivate of the pET32a( +) expression vector (Millipore).

The pET-K2 vector lacking the thioredoxin fusion sequence and with a kanamycin resistance was prepared in two steps. Firstly, the pET-K1 vector was constructed by ligating T4 kinase-treated 4481 bp and 825 bp PCR fragments that were amplified as follows: The former PCR fragment was amplified on the pET-32a expression vector template (Millipore) with the P29FOR–P30REV primers (Table S1). The latter PCR fragment was amplified on a pCambia1304 vector template (Roberts et al. 2002) with P31FOR–P32REV primers (Table S1). The final pET-K2 vector was prepared by PCR amplifying 5251 bp DNA fragment on the pET-K1 vector template with the P33FOR–P34REV primers (Table S1). The obtained PCR amplicon was treated with T4 polynucleotide kinase and ligated.

The construct pET-K2*DbChitI-3*^S−^ was verified by sequencing and introduced into *E. coli* BL21-CodonPlus (DE3) RIL expression strain (Agilent). The expression of recombinant protein rDbChitI-3^S−^His was induced by adding 1 mM IPTG to the bacterial culture at OD_600_ of 0.6 and followed by incubation at 37 °C for 3 h. The cells were collected by centrifugation at 4 °C and stored at – 80 °C.

The confirmation of rDbChitI-3^S−^His expression was performed on 12% (w/v) SDS-PAGE using total cell protein extracts from the bacterial pellet (1 mL of bacterial culture) of induced and non-induced *E. coli* culture carrying recombinant plasmids. Before loading on the gel, harvested cells were resuspended in sample buffer [45 mM Tris–HCl, (pH 6.8); 10% (v/v) glycerol; 1% (w/v) SDS; 0.01% (w/v) bromophenol blue; 50 mM DTT].

Isolation and purification of rDbChitI^−S^His protein were performed from 50 mL of bacterial culture while following the centrifugation; the pellet was resuspended in 5 mL of SDS lysis buffer [50 mM NaH_2_PO_4_; 300 mM NaCl; 10 mM imidazole; (pH 8.0)], then supplemented with SDS to final concentration 2% (w/v) and treated as described by Rajninec et al. (2020). The supernatant was collected and loaded on Ni–NTA agarose column (Qiagen) pre-equilibrated with lysis buffer without SDS. Unbound proteins were removed with 20 mL of wash buffer [50 mM NaH_2_PO_4_; 300 mM NaCl; 20 mM imidazole (pH 8.0)]. Loading and washing steps were carried out at 8 °C, and precipitated SDS was removed. The rDbChitI^−S^His protein was eluted at room temperature using (3 × 0.5 mL) elution buffer [50 mM NaH_2_PO_4_; 300 mM NaCl; 250 mM imidazole (pH 8.0)].

Eluted fractions were pooled and imidazole was removed using the Econo-Pac 10DG Columns (Bio-Rad). The total cell proteins of induced and non-induced bacterial culture and purified rDbChitI-3^S−^His protein were analyzed on 12% (w/v) SDS-PAGE. Detection of enzyme activity was performed as follows: The same protein samples separated on 12% (w/v) SDS-PAGE gel containing 0.01% (w/v) glycol chitin prepared by acetylation of glycol chitosan (Sigma-Aldrich) as described by Trudel and Asselin (1989) were re-naturated in the solution containing 50 mM sodium acetate (pH 5.2) and 1% (v/v) Triton X-100 overnight, then followed incubation in 50 mM sodium acetate (pH 5.2) for 2 h, rinsing with distilled water and detection of bands with chitinase activity as dark zones after staining the gel with 0.01% (w/v) Fluorescent Brightener 28 for 15 min and UV illumination.

### Determination of chitinase activity

Acetic acid-swollen FITC-chitin (N-fluorescein-labelled chitin) was prepared according to Tikhonov et al. (2004) with a modification involving a 2-h treatment of prepared substrate in 10% acetic acid, followed by thorough washing with sterile double-distilled water. Chitinase activity assay was performed in a reaction mixture (1 mL) containing 100 mM citrate–phosphate buffer (McIlvaine buffer), acetic acid-swollen FITC-chitin substrate (100 µL) and purified rDbChitI-3^S−^His protein (17 µg). The enzymatic reaction was kept in a thermo-shaker at selected temperatures for 60 min. Following centrifugation for 15 min at 16 000 *g*, 100 µL of the sample was mixed with 900 µL of 0.5 M Tris–HCl (pH 8.9), and 100 µL of the mixture was transferred into 96-well plates. Released fluorescence was measured on a Synergy H1 microplate reader (BioTek) using 483/528 nm excitation and emission filters. Chitinolytic activity values were expressed as an average of the relative fluorescence units normalized to the blank (reaction mixture with heat-inactivated protein).

The influence of pH on the hydrolytic activity of recombinant chitinase involved the following buffers (each 100 mM): citrate–phosphate (2.2, 2.5, 3.0, 3.6, 4.0, 4.5, 5.0, 5.5, 6.0, 6.5, 7.0, 7.5 and 8.0), glycine–HCl (pH 2.2, 2.5, 3.0 and 3.6) and sodium acetate (3.6, 4.0, 4.5 and 5.0). Temperature optimum was assessed by incubating the chitinase in citrate–phosphate buffer (pH 2.5) at constant temperature ranging from 10 °C to 50 °C. Overall, three biological replicates were analyzed. Residual activity was expressed as the percentage of activity value compared to the highest activity value.

The stability of the rDbChitI-3^S−^His enzyme was tested at 25 °C and 37 °C. Pre-incubation of the rDbChitI-3^S−^His protein (17 µg) at tested temperature for 14 h was followed by supplementation with 100 µg substrate and subsequent enzyme reaction for 1 h. As a control, enzyme reaction involving rDbChitI-3^S−^His enzyme without the pre-incubation step was used.

Substrate specificity for chitin powder (from shrimp shells, Sigma-Aldrich) and glycol chitin (Trudel and Asselin 1989) was determined by incubating the purified rDbChitI-3S-His (33.8 µg) protein with each polysaccharide in 1 mL of 100 mM citrate–phosphate buffer (pH 2.5) for 24 h. The measurement of soluble carbohydrates products of enzyme reaction was performed using the 3,5-dinitrosalicylic acid (DNS) method (Shrestha et al. 2011). 4-Methylumbelliferyl N-acetyl-β-D-glucosaminide (4-MUF-NAG) (Sigma-Aldrich), 4-methylumbelliferyl N,N′-dicetylchitobioside [4MU-(GlcNAC)_2_] (Sigma-Aldrich) and 4-methylumbelliferyl β-D-N,N′,N´´-triacetylchitotrioside [4MU-(GlcNAC)_3_] (Sigma-Aldrich) substrate specificity was determined using the fluorometric chitinase assay kit (Sigma-Aldrich). Each reaction mixture (100 µL) contained 2.5 µL substrate (20 mg/mL) in 100 mM citrate–phosphate buffer (pH 2.5) and 8.6 µg (250 µmol) purified rDbChitI-3^S−^His protein and was incubated at 25 °C for 1 h. Commercial chitinase from *Streptomyces griseus* (Sigma-Aldrich) was used as a control. The reaction mixture consisted of 2.5 µL of substrate (20 mg/mL) in 100 mM citrate–phosphate buffer (pH 6.0), along with 7.5 µg (250 µmol) of chitinase and corresponding substrate. The reaction was conducted at 37 °C for 1 h. Chitinolytic activity values were expressed as an average of the relative fluorescence units normalized to the blank (reaction mixture with heat-inactivated protein). Values represent the mean of three biological replicates from individual protein isolations.

### Gene expression analysis using real-time quantitative PCR (RT-qPCR)

Total RNA was isolated from different organs (stem, root, leaf blades, petioles and flowers) of non-treated *D. binata* plants and leaf blades treated by different inducers (Bekesiova et al. 1999). The residual genomic DNA was removed by RNase-free DNaseI treatment (Thermo Fischer Scientific). First-strand cDNA synthesis was performed using the Maxima H Minus First Strand cDNA Synthesis Kit (Thermo Fischer Scientific) according to the manufacturer's instructions. Total RNA (1–2 µg) was converted to cDNA in a 20-µL reaction mixture containing 5 µM oligo(dT)18 primer and 1 µL Maxima H Minus enzyme mix. The mixture was incubated at 50 °C for 30 min, and the reaction was terminated by incubation at 85 °C for 5 min. Real-time PCR was performed using a LightCycler Nano (Roche Applied Science). Before selecting the final reference gene, the expression stability of three candidate reference genes (β-actin, α-tubulin and elongation factor 1-α) was tested in experiments with intact plant tissues and leaves treated with different compounds. Based on the M values of geNorm (Vandesompele et al. 2002), actin was selected as the most suitable reference gene. The PCR contained Luminaris Color HiGreen qPCR Master Mix (Thermo Fischer Scientific) mixed with 1 µL of cDNA (diluted 1:4) per 10 µL reaction volume, each containing 0.3 µM P23FOR-P24REV (*D. binata* actin, MN481116) (Jopcik et al. 2017) and P25FOR-P26REV (MN481117 *D. binata* chitinase) primers (Table S1). The reaction was initiated by a uracil-DNA glycosylase step at 50 °C for 2 min, followed by one cycle at 95 °C for 10 min and 40 cycles at 95 °C for 15 s, 60 °C for 60 s, followed by a melting curve analysis step to confirm the specificity of the amplified products. Four independent biological with two technical replicates were performed for each treated and non-treated sample.

One-way ANOVA was used to reveal statistically significant differences in gene expression at *P* < 0.05, followed by the posthoc Tukey test for pairwise comparisons with the control group (non-treated leaf blades). In the case of two experimental groups, we relied on an unpaired t-test. Analysis was done in GraphPad Prism version 9.2.

## Results

### Cloning and sequence analysis of the *DbChitI-3* gene and deduced amino acid sequence

**Fig. 1:**
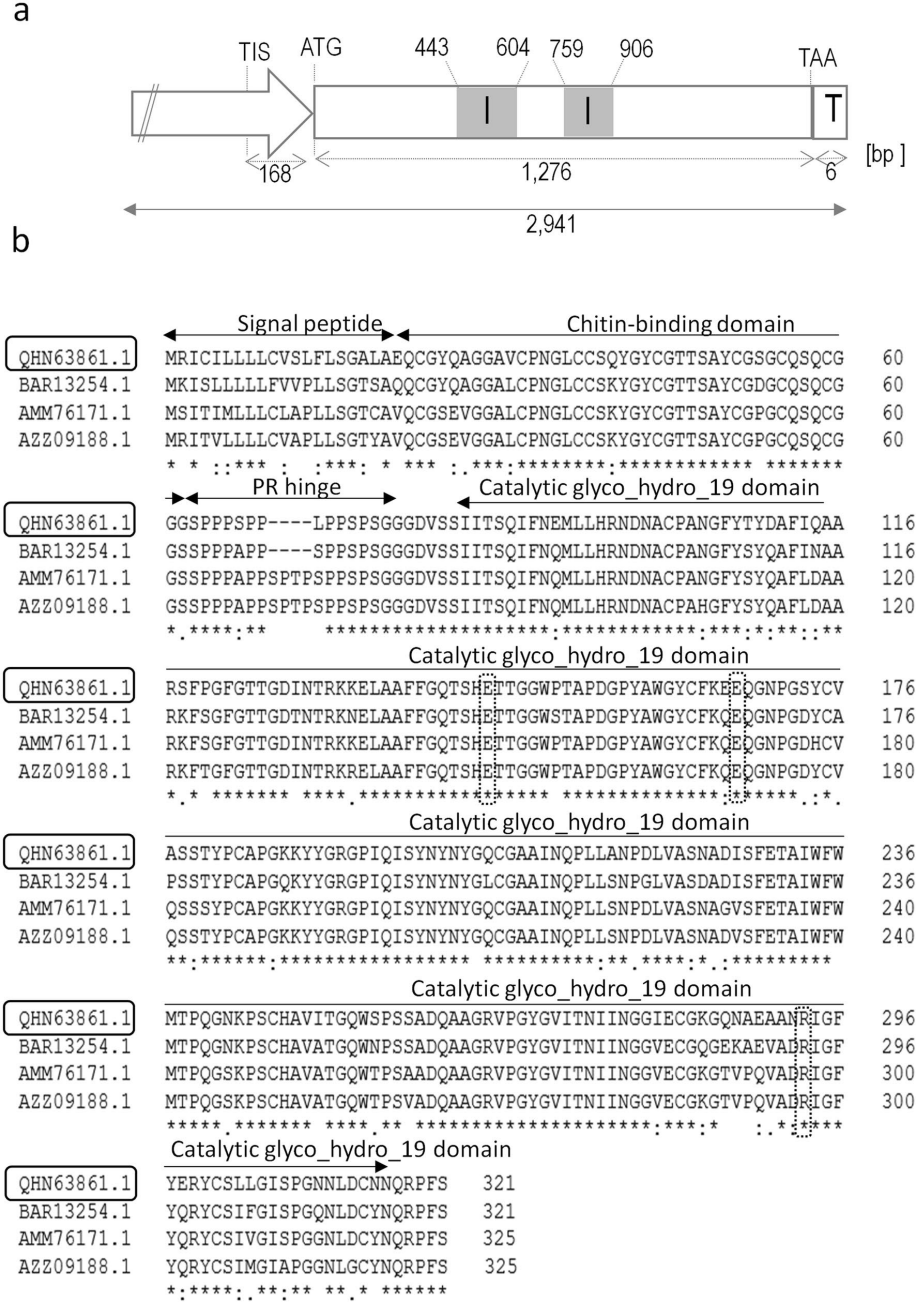
The *DbChitI-3* gene sequence characterization. **a** Schematic representation of the *DbChitI-3* gene. The transcription start site was experimentally confirmed 168 bp upstream of the ATG. Within the gene, two introns (I) were confirmed. A putative polyA signal was identified six nucleotides downstream of the TAA stop codon. TIS, transcription initiation start. **b** Deduced DbChitI-3 amino acid sequence and homology analysis of the chitinases from *Drosera* species [*D. binata* DbChitI-3 (QHN63861.1), *D. adelae* (BAR13254.1), *D. rotundifolia* (AMM76171.1) and *D. capensis* (AZZ09188.1)]. The sequences for signal peptide (1–20), chitin-binding domain (20–61), proline-rich (PR) hinge (63–75), and catalytic glyco_hydro_19 domain (93–314) are indicated by double-sided arrows. The amino acid residues in interrupted boxes are essential for catalysis

A homology-based strategy was used to isolate the chitinase gene from the carnivorous plant *D. binata*. The DNA fragment of a length of 868 bp of the conserved genomic region of class I chitinases was amplified with *D. rotundifolia* chitinase specific primers (KU516826.1) (Jopcik et al. 2017) and Synergy DNA polymerase recommended for low specificity PCR. Subsequently, a genome walking approach was used to amplify the 5' and 3' overlapping gene fragments of 2141 bp and 433 bp, respectively. A 2941 bp contig of genomic DNA was obtained from the overlapping sequences of the PCR fragments and designated as *DbChitI-3*. Homology search using the BLASTx and NetGene2 algorithms revealed the presence of a chitinase gene 1276 bp in length, including the putative ATG start and TAA stop codons for translation. The 5' RACE and 3' RACE analyses experimentally defined the full-length cDNA of *DbChitI-3*, whereas the start of transcription and the transcription termination were 168 bp upstream of the ATG start codon and six bp downstream of the TAA stop codon (Fig. 1a).

Next, analysis using the NetGene2 program predicted the presence of two introns of 162 and 148 bp in length with splice sites TG/GA and TA/CA. Splicing of the intron was confirmed by aligning the DNA sequence *DbChitI-3* with the corresponding coding sequence (*cdsDbChitI-3*) obtained after RNA isolation from leaves, cDNA synthesis, and PCR with the P21FOR–P22REV primers. The genomic clone *DbChitI-3*, the corresponding *cdsDbChitI-3*, and the full-length cDNA of the *DbChitI-3* clone were deposited in GenBank under accession number MN481117.1. The amino acid sequence of *cdsDbChitI-3* was designated QHN63861.1.

The isolated genomic clone of *DbChitI-3* also contained 1414 bp of DNA sequence upstream of the experimentally defined transcription initiation start (TIS). Since in silico analysis (Table S2) revealed no TATA element within the DNA, approximately – 40 nucleotides upstream of TIS (Butler et al. 2002), we suggest that the *DbChitI-3* promoter may belong to the group of TATA-less promoters. The sequence of ~ 1000 nucleotides upstream of TIS was characterized by a higher content of A + T bases (65%), similar to other plant promoters (Bhat et al. 2014). The major determinant of promoter efficiency, the CAAT box, which acts in two directions (Porto et al. 2014), was detected at positions – 153( +), – 7(-), + 18( +), + 60( – ), + 137( +) nucleotides near TIS.

Next, the bioinformatic analysis revealed the presence of two main groups of putative *cis*-acting elements. The first group includes light- and tissue-specific motifs represented by GATA (*n* = 6), GT1(*n* = 14), CIACADIANLELHC (*n* = 3), TBOX (*n* = 2), POLLEN1LELAT52 (*n* = 7), GTGANTG10 (*n* = 7), ROOTMOTIF (*n* = 5), RAV1 (*n* = 1). The latter included putative *cis-*regulatory elements responsive to hormones and biotic and abiotic stresses [EBOX (*n* = 6), MYB (*n* = 6), ACGTATERD1 (*n* = 2), ARR1AT (*n* = 9), WBOX (*n* = 9)]. The role of WBOX- and EBOX *cis*-elements in the chitinase promoters were previously described as mediating chitin elicitor- and jasmonic acid responsiveness, respectively, during defence against pathogens (Chujo et al. 2009; Miyamoto et al. 2012). The chitin in non-carnivorous plants elicits a defence response; in the leaves of carnivorous plants, it indicates the presence of prey for digestion (Jopcik et al. 2017).

The open reading frame of *DbChitI-3* consisted of 966 nucleotides encoding 321 amino acid residues (Fig. S1). The program SignalP indicated that the first 20 amino acids sequence represented a putative signal peptide for transport to the endoplasmic reticulum (ALA-EQ D = 0.958 D-cutoff = 0.450). The remaining 301 amino acids formed a mature protein with an approximate molecular mass of 31.71 kDa and a theoretical isoelectric point of 5.35 (Fig. S1).

The INTERPRO Search software predicted that the encoded protein DbChitI-3 is a chitinase with a glyco_hydro_19 domain (IPR000726) from position 83 to 314. In addition, a chitin-binding domain (IPR001002) and a lysozyme-like superfamily domain (IPR023346) were detected at positions 20–61 and 79–320, respectively. The amino acid sequence of DbChitI-3 showed strong homology to the class I chitinases of other *Drosera* species [*Drosera adelae* (BAR13254.1), *Drosera capensis* (AZZ09188.1), *D. rotundifolia* (AMM76171.1)], reaching more than 80% identities (Fig. 1b). The composition of the remaining amino acids affected the theoretical pI while *D. binata* and *D. adelae* had a calculated acidic pI of 5.33 and 5.56, respectively, and differing from *D. capensis* and *D. rotundifolia* with basic pI of 7.49 and 7.88, respectively. All these chitinases contain the SHETT consensus sequence, which is highly conserved for all glyco_hydro_19 chitinases. Considering *Brassica juncea* class I chitinase (GenBank accession number AAF02299.1) as the reference structure for the catalytic domain (Ubhayasekera 2011), the catalytically important triad in DbChitI-3 is represented by amino acid residues E145, E167, and R293 (Fig. 1b). The protein is extracellularly targeted because it lacks the C-terminal extension sequence (Eilenberg et al. 2006). The GlycoEP program, with a defined threshold of 0.5, failed to detect potential N-linked glycosylated sites for amino acid residues within the mature chitinase sequence (lacking signal protein). Potential phosphorylation sites were also examined using the NetPhos program. A total of 17, 8, and 5 phosphorylation sites were detected for serine, threonine, and tyrosine, respectively, with the same threshold as in the previous in silico analysis.

**Fig. 2:**
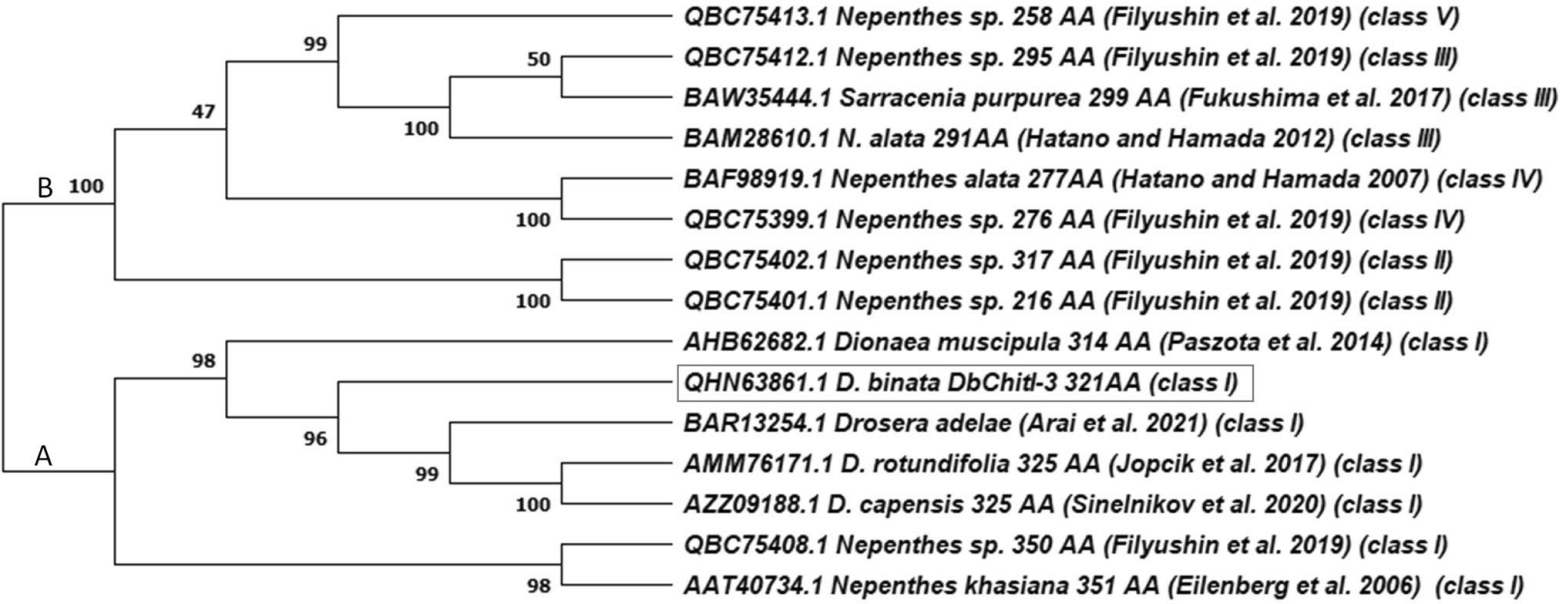
Phylogenetic tree of DbChitI-3 and other chitinases from carnivorous plants. The unrooted tree was constructed using the Neighor-Joining method with the program MEGA X. The reliability of interior branches was assessed with 1000 bootstrap resamplings. The GenBank accession numbers, the corresponding species, the number of amino acids (AA) with the corresponding reference, and the chitinase class are indicated in the tree

The classification of the DbChitI-3 protein in class I chitinases also confirmed phylogenetic profiling performed with members of the extracellular chitinases of carnivorous plants available in the NCBI database. As shown in Fig. 2, DbChitI-3 was clustered together with the amino acid sequences of the carnivorous chitinases containing the Glyco_hydro_19 and chitin-binding domains (class I). The other chitinases with a single domain, either the glyco_hydro_19 (class II and IV) or the glyco_hydro_18 (class III and V), were grouped in cluster B.

### Determination of enzyme activity of purified chitinase protein

**Fig. 3:**
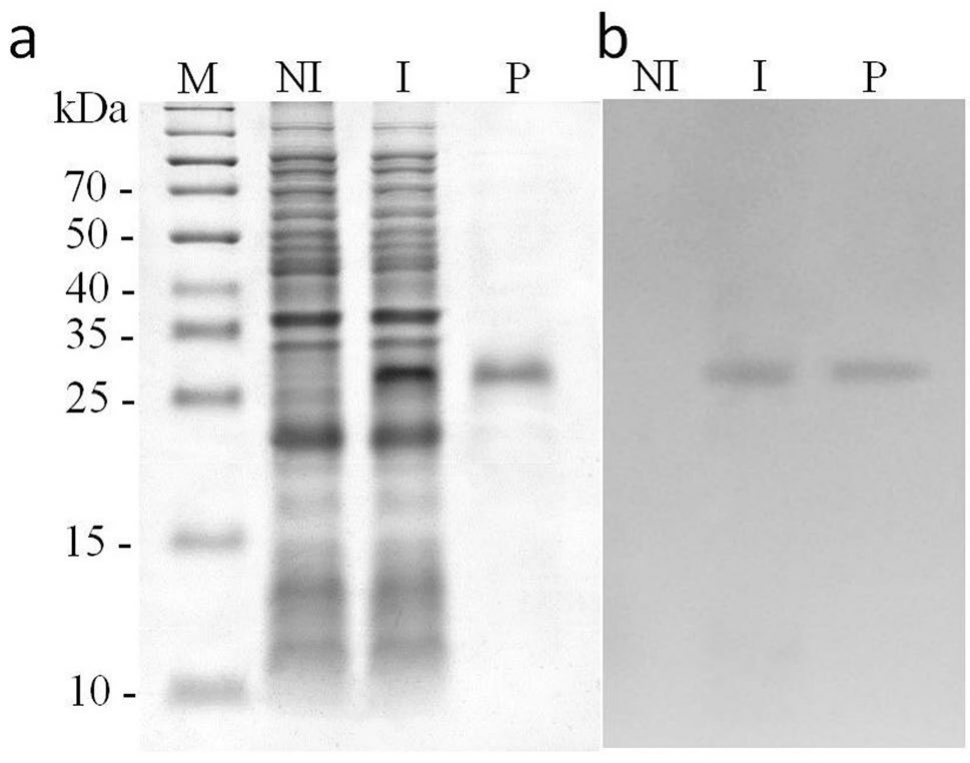
Purification and enzyme activity of rDbChitI-3^S−^His protein. **a** SDS-PAGE analysis of the rDbChitI-3^S−^His protein expressed in *E. coli* and isolated using the His-Tag purification system. **b** Zymogram of chitinase activity. The samples without heating were separated on 12% (w/v) SDS gel containing 0.01% glycol chitin under non-reducing conditions and then renaturated in the solution containing 1% (v/v) Triton X-100. The bands with chitinase activity were detected as dark zones after staining the gel with 0.01% (w/v) Fluorescent Brightener 28. Lanes: M, Spectra Multicolor Broad Range Protein Ladder (Thermo Fischer Scientific); NI, total cell proteins from non-induced *E. coli* BL21-CodonPlus(DE3)RIL/pETK2rDbChitI-3^S−^ I, total cell proteins from induced *E. coli* BL21-CodonPlus(DE3)RIL/pETK2rDbChitI-3^S−^His*;* P*,* rDbChitI-3^S−^His protein after purification on Ni–NTA agarose

To assess the chitinase activity and enzymatic parameters of the DbChitI-3 protein, we produced it recombinantly in *E. coli* as a fusion with a 6xHis tag sequence at both the N- and C-termini (rDbChitI-3^S−^His, Fig. 3). Following IPTG induction, the bacterial culture yielded a recombinant protein with a molecular mass of 33.8 kDa. Subsequently, after purification on Ni–NTA agarose (Fig. 3a) we confirmed the capacity of rDbChitI-3S-His to hydrolyze the glycol chitin substrate using the zymogram method within polyacrylamide gel (Fig. 3b).

**Fig. 4:**
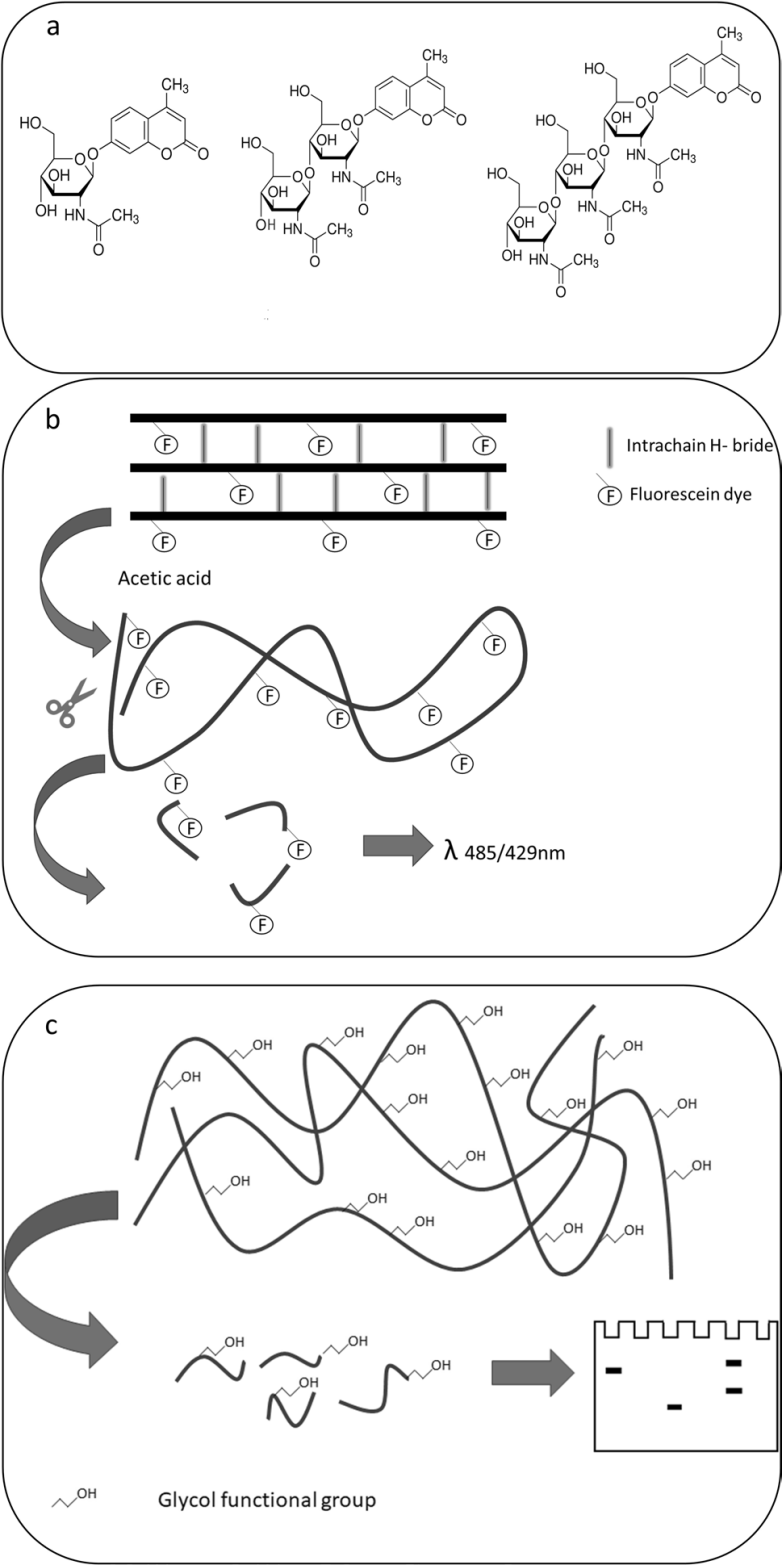
Substrate specificity of the rDbChitI-3S-His protein. **a** The recombinant chitinase shows no enzymatic activity on the substrates 4-MUF-NAG, 4MU-(GlcNAC)_2_ and 4MU-(GlcNAC)_3._ **b** The enzyme exhibits very limited activity on FITC-labelled chitin powder but cleaves acetic acid-swollen chitin, as assessed through the fluorometric assay. **c** The rDbChitI-3^S−^His protein is able of breaking down glycol chitin, as detected by gel electrophoresis and the DNS method

To characterize the rDbChitI-3S-His enzymatic activity in more detail we used a range of mono-, oligo- and polysaccharide substrates. Fluorometric assays revealed that the rDbChitI-3S-His enzyme did not cleave the 4-MUF-NAG, 4MU-(GlcNAC)_2_ and 4-MU-(GlcNAC)_3_ substrates, indicating a deficiency in β-N-acetylglucosaminidase, chitobiosidase, and short chitooligomer-specific endochitinase activity, respectively (Fig. 4a).

Subsequently, we conducted experiments to evaluate the efficacy of the rDbChitI-3S-His enzyme in cleaving various long-chain N-acetylglucosamine substrates. Glycol chitin, also known as N-acetylated glycol chitosan, which is more water-soluble compared to chitin, was subjected to degradation by the rDbChitI-3S-His enzyme, as evidenced by zymogram analysis (Fig. 3b) and confirmed by DNS assay. A highly sensitive fluorometric assay further demonstrated the enzyme's low efficiency in cleaving FITC-chitin (high molecular weight chitin labelled by one FITC molecule approximately per 45 glucosamine residues). Conversely, the enzyme exhibited high efficiency in cleaving the same high molecular weight FITC-chitin after it was treated with acetic acid to induce swelling, which partially disrupted intrachain hydrogen bonds (Fig. 4). These results suggest that the rDbChitI-3S-His enzyme is incapable of digesting intact chitin.

**Fig. 5:**
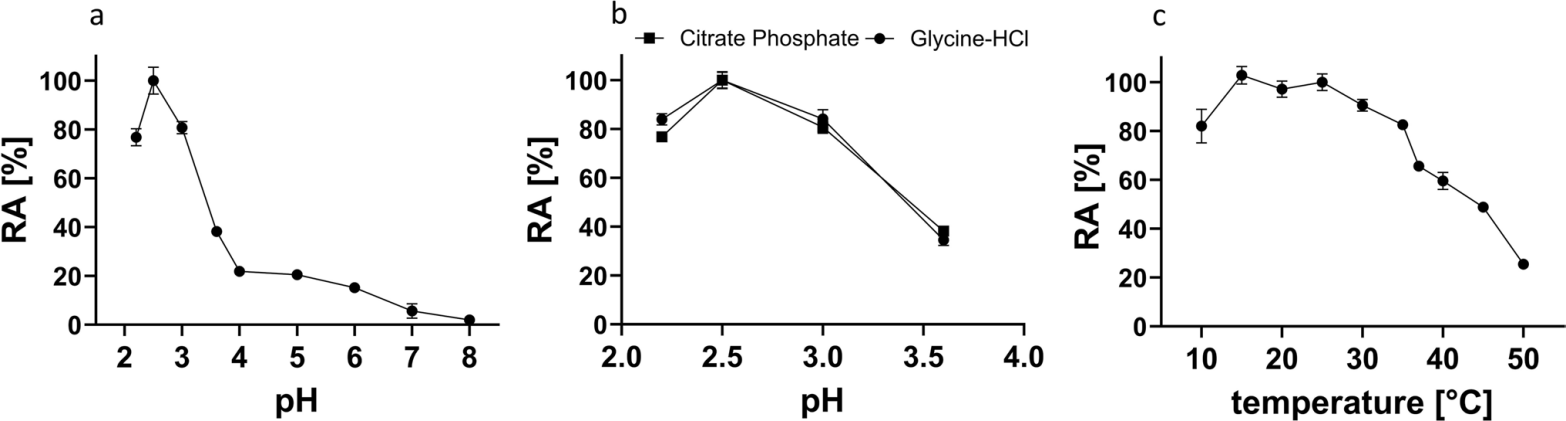
The effect of pH, buffer conditions and temperature and on the enzyme activity of the purified rDbChitI-3.^S−^His protein. **a** Enzyme activity detected in 100 mM citrate–phosphate buffer in pH range from 2.2 to 8.0 **b** The pH values were adjusted with the following buffer systems: glycine–HCl and citrate–phosphate (pH 2.2–3.5). **c** The temperature optimum was tested in the range from 10 °C to 50 °C. The enzyme activity was measured after 1 h incubation with the acetic acid-swollen FITC-chitin substrate. Residual activity (RA) was expressed as the percentage of activity value compared to the highest activity value. Error bars depict standard errors (*n* = 3)

Consequently, the fluorometric assay with acetic acid-swollen FITC-chitin as a substrate was selected as an optimal method to assess biochemical characteristics of the the rDbChitI-3^S−^His protein (Fig. 5). Firstly, we determined the activity of the purified enzyme with a pH range tested from 2.2 to 8.0. As shown in Fig. 5a, the highest enzyme activity was detected in 100 mM citrate–phosphate buffer in the pH range from 2.2 to 3.0, followed by a steep decrease in activity, with less than 50% of activity detected at pH levels higher than 3.5. A similar trend of enzyme activity was observed in glycine–HCl buffer (pH 2.2–3.6) (Fig. 5b). Next, the temperature range for enzyme activity of the rDbChitI-3S-His protein was evaluated. The optimal temperature spanned from 15 °C to 30 °C, with 82% of activity retained at 10 °C. Conversely, temperatures over 35 °C had an obviously negative effect on enzyme activity, with 64% and 25% activity retained at 37 °C and 50 °C, respectively (Fig. 5c). Exposing the tested enzyme to a 14 h pre-induction at 25 °C and 37 °C without substrate, followed by subsequent enzyme activity testing using acetic acid-swollen FITC-chitin for 1 h, revealed the evident stability of rDbChitI-3S-His enzyme, with only a 25% decline in activity compared to the adequate control without the pre-incubation step.

Finally, to compare the enzymatic parameters with a commercial chitinase from *Streptomyces griseus*, a fluorometric assay using acetic acid-swollen chitin as a substrate was conducted under optimal conditions for both enzymes (pH 2.5, at 25 °C for rDbChitI-3S-His, and pH 6.0, at 37 °C for *S. griseus* chitinase), employing equimolar amounts of enzymes (250 µmol). This resulted in the release of 41,144.5 ± 1878 RFU and 63,946.3 ± 2054 RFU of fluorescein from acetic acid-swollen FITC-chitin over 30 min, respectively. These findings suggest that the efficiency of rDbChitI-3S-His reaches 64.3% of that of *S. griseus* chitinase under optimal conditions.

### Expression profile of the *DbChitI-3* gene in different plant organs and upon application of various compounds mimicking insect prey presence

**Fig. 6:**
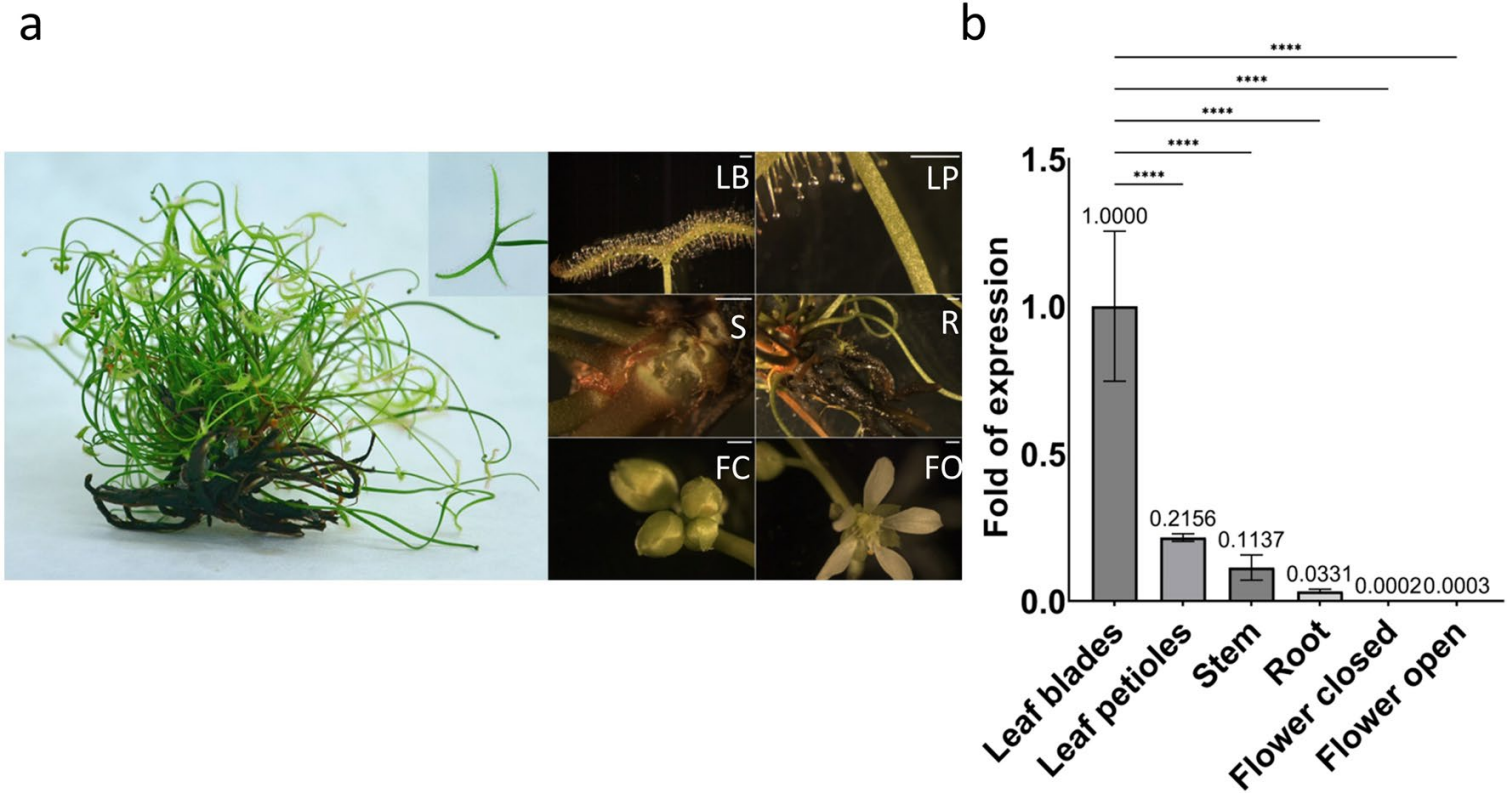
*Drosera binata* plants and gene expression profile of *DbChitI-3* gene. **a** *Drosera binata* plant with a detailed image of the leaf blade (LB), leaf petiole (LP), stem (S), root (R), flower closed (FC) and flower opened (FO); scale bar represents 1 mm. **b** Relative *DbChitI-3* mRNA transcripts level in individual organs. Gene expression in intact leaf blades was evaluated as a basal expression, referred to as 1. Data from RT-qPCR were normalized relative to the abundance of the endogenous control gene β-actin mRNA transcripts. Error bars depict standard errors (*n* = 4). The difference is significant at ****P < 0.0001 compared to the control (leaf blades)

In order to assess if the expression of the *DbChitI-3* gene is organ-specific, we analyzed its mRNA transcription level in roots, stem, leaf blade, petiole, open flower and closed flower of non-treated, in vitro-grown *D. binata* plants (Fig. 6a). RT-qPCR quantified the *DbChitI-3* gene transcripts, while β-actin transcription was used for normalization. In non-treated plants, *DbChitI-3* gene expression was detected in leaf blades; it was significantly lower in petioles and stem, reaching a 5- and ninefold decrease in mRNA levels, respectively. Almost no transcription of the *DbChitI-3* gene was detected in roots and flowers (Fig. 6b).

**Fig. 7:**
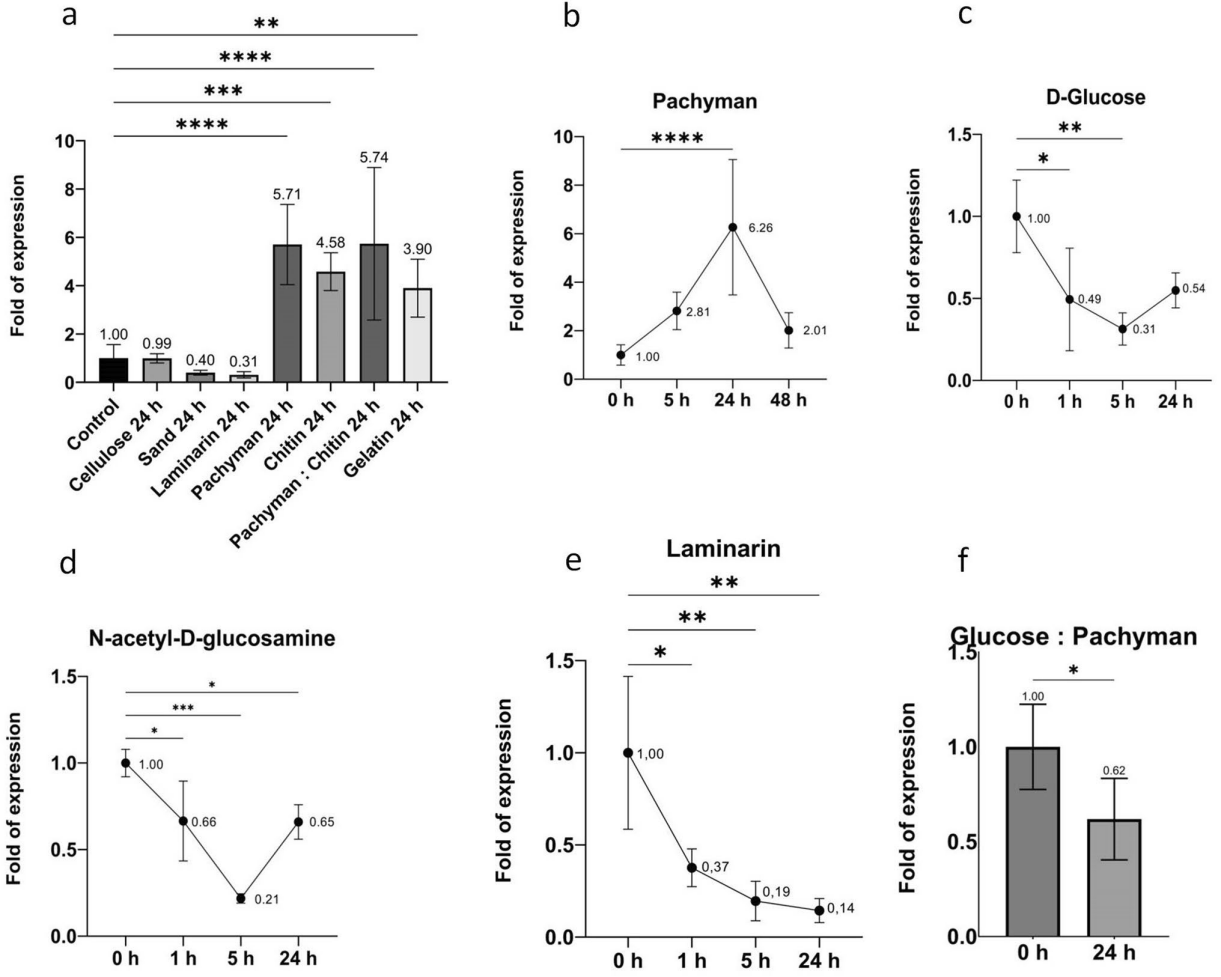
The *DbChitI-3* gene expression profile upon application of different compounds on the leaves. **a** The *DbChitI-3* mRNA expression level in leaf blades upon application of different potential inducers after 24 h; the basal expression refers to non-treated control leaves, referred to as 1. **b-f** Time course of *DbChitI-3* mRNA expression level after application of strong inducer pachyman (**b**), monosaccharides 100 mM D( +)-glucose (**c**), 100 mM N-acetyl-D-glucosamine (**d**), 2% laminarin (**e**) and combination of glucose and pachyman (**f**). Data from RT-qPCR were normalized relative to the abundance of the endogenous control gene β-actin mRNA transcripts. Error bars depict standard errors (*n* = 4). The difference is significant at *P < 0.05, **P < 0.01, ***P < 0.001, ****P < 0.0001 compared to the control

The glandular trichomes of the leaf blades produce hydrolytic enzymes involved in the digestive processes of *D. binata* plants, while it has been postulated that the digestion processes should be fine-tuned to ensure a cost–benefit ratio. In order to elucidate how the *DbChitI-3* gene is regulated, we tested its mRNA transcription level in leaves after treatment with a variety of compounds for 24 h (Fig. 7).

First, we tested a specific substrate for chitinase, crustacean chitin, as in silico analysis indicated within the promoter, the WBOX *cis*-regulatory elements that are required for chitin elicitor-responsive expression via WRKY transcriptional factors in plant cells (Chujo et al. 2009). Then, some other compounds–potential sources of nutrients such as pachyman (fungal β−1,3-glucan), cellulose (β−1,4-glucan), gelatin and sand (all in solid form) were tested as well. The leaves of *D. binata* collected before applying the potential inducer were used as a control. A notable up-regulation of the transcription of the *DbChitI-3* gene was observed with the chitin (4.6-fold) and the proteinaceous compound gelatin (3.9-fold). Surprisingly, pachyman (β−1,3-glucan), not substrate for chitinases, showed the highest (5.7-fold) increase in relative mRNA transcription abundance of the tested gene. The mixture of pachyman:chitin did not result in an additive effect of the two potent inducers when the 5.7-fold increase in basal mRNA *DbChitI-3* gene transcripts after 24 h was equivalent to the application of pachyman alone, probably reaching expression maximum of the analyzed gene under defined conditions (Fig. 7a).

Then we attempted to answer whether another β−1,3-glucan–laminarin with a lower degree of polymerization (DP ~ 20–30) has the same effect on the transcription of the *DbChitI-3* gene as pachyman (DP ~ 255). Both polymers were applied in solid form to the leaf blades for 24 h. As shown in Fig. 7a, only pachyman up-regulated the mRNA transcription of the *DbChitI-3* gene, suggesting that the structure of the β−1,3-glucan (degree of polymerization) affects the expression of the chitinase involved in the digestion process.

Finally, we have found that cellulose had no effect on basal *DbChitI-3* gene expression; sand and laminarin applied to leaves decreased chitinase gene expression level, reaching only 40% and 31% mRNA *DbChitI-3* transcripts level of the non-treated leaves (Fig. 7a).

The time course of the *DbChitI-3* mRNA abundance after applying the strongest inducer–pachyman on the leaves showed its maximum 24 h after application, followed by a gradual decrease in expression activity (Fig. 7b).

We then asked how the hydrolysis products of both polysaccharides (chitin and pachyman) affect the *DbChitI-3* gene expression. The D( +)-glucose and N-acetyl-D-glucosamine were added as 100 mM solutions for 1 h, 5 h and 24 h on the leaves. Both compounds significantly decreased *DbChitI-3* mRNA transcription compared to baseline levels of mRNA transcripts, with a minimum of 31% for D( +)-glucose and 21% for N-acetyl-D-glucosamine, reached 5 h after application (Fig. 7c,d). When a lower concentration of D( +)-glucose (10 mM) was tested, no inhibitory effect of *DbChitI-3* basal expression on mRNA levels was detected (Fig. S2). Laminarin applied as a 2% (w/v) solution also caused the repression *of DbChitI-3* gene transcription, while its expression dropped to 14%, 24 h after treatment (Fig. 7e), probably due to glucose units released by present glucanases in the digestive fluid.

Finally, we tested the effects of the combination of strong inducer and suppressor on the transcriptional activity of the *DbChitI-3* gene. Plants were sprayed with a 100 mM glucose solution, followed by pachyman powdering and analyzed 24 h after application. Compared to the control, we observed a decrease in *DbChitI-3* mRNA transcripts to 60% of basal transcriptional activity, indicating that regulation via available nutrients dominates the transcriptional activity of genes for digestive enzymes (Fig. 7f).

## Discussion

This study identifies a unique class I chitinase, DbChitI-3, from *Drosera binata*, expanding the known repertoire of chitinases in the genus of *Drosera*, and provides insights into its enzymatic properties, substrate specificity, and role in prey digestion within acidic environments.

Mechanical irritation or chemical stimulation of *D. binata* plants leads to the activation of their sticky flypaper traps – mucilage-producing tentacles that bend toward a stuck prey that is subsequently killed and digested (Poppinga et al. 2013). In species of *Drosera*, proteins are preferred as a nitrogen source when digesting insect prey, although the hydrolysis and uptake of nitrogen from chitin have also been demonstrated (Pavlovic et al. 2016). For this purpose, extracellular chitinases of several classes can be harnessed, although only a few have been detected in the digestive fluids of individual *Drosera* species. The participation of class I chitinase in digestion has been described in plants of *D. rotundifolia* (Jopcik et al. 2017), *D. adelae* (Arai et al. 2021) and *D. capensis* (Pavlovic et al. 2016). The present study adds chitinase from *D. binata* to this list. Transcriptome and proteome studies of sundews, pitcher plants and Venus flytrap have revealed that chitinases of class I, III and IV are expressed in the digestive organs of these carnivorous plants (Rottloff et al. 2011; Hatano and Hamada 2012; Paszota et al. 2014).

Chitinases class I isolated from *Drosera* species (Fig. 1b) share the same gene structure, and the translated amino acid sequences show more than 80% similarity, with the greatest variability found in the length of the proline-rich hinge. Extracellular targeting is characteristic of all identified *Drosera* spp. class I chitinases and is thought to have changed their role from defensive to carnivorous (Schulze et al. 2012; Wheeler and Carstens 2018). In silico analysis revealed that the mature DbChitI-3 protein has an acidic theoretical pI based on its amino acid composition, similar to the chitinase of *D. adelae.* In contrast, the chitinases of *D. rotundifolia* and *D. capensis*, which participate in digestion, exhibit a slightly basic pI. The importance of chitinases’ basic or acidic character in the decomposition of insect prey is still unclear. Despite sharing the same protein structure and having over 80% amino acid identity, significant differences were observed in the enzymatic parameters and substrate specificity between *D. binata* chitinase (DbChitI-3) and *D. rotundifolia* chitinase (DrChit) (Rajninec et al. 2020). The former exhibited maximum enzyme activity at pH 2.5 and temperatures ranging from 15 °C to 30 °C, while the latter showed optimal activity at pH 6.0 and 38 °C.

The acidity of digestive fluids is a defining feature of carnivorous plants, playing a key role in prey digestion and nutrient adsorption. While the pH of the digestive fluid in *D. binata* and *D. rotundifolia* has not been measured, studies of other *Drosera* species provide some insights. In *D. capensis*, the digestive fluid pH ranges from 2.5 to 5.0, while in *D. adelae*, the lowest recorded value is 4.3 (Freud et al. 2022). Similarly, in *Dionea muscipula,* pH values range from 1.5 to 5.0, depending on the treatment. Acidic conditions are crucial for the activity of digestive enzymes, such as chitinases and proteases, which ensure optimal prey decomposition (Bazile et al. 2015; Buch et al. 2015; Saganova et al. 2018). Variations in pH and enzymatic activity between species likely reflect their unique ecological adaptations and prey-processing strategies (Adlassnig et al. 2011).

The acidic DbChitI-3 chitinase, identified in the digestive processes of the carnivorous plant *D. binata*, is comparable to the chitinases found in the stomachs or intestines of various omnivorous animals, including mice, pigs, chickens, and common marmosets (Ohno et al. 2016; Tabata et al. 2019). However, unlike *DbChitI-3*, animal acidic chitinases belong to the glycoside hydrolase GH18 family (Bussink et al. 2007). These chitinases, which have a distinct evolutionary origin, likely evolved in certain omnivores as digestive adaptations to process dietary chitin (Tabata et al. 2022).

Notably, DbChitI-3 exhibits distinct substrate preferences in enzymatic reactions. While *D. rotundifolia* chitinase cleaved chitin powder (Rajninec et al. 2020), *D. binata* chitinase digested only acidic acid-swollen chitin. This suggests that the involvement of the DbChitI-3 enzyme in the digestive processes of *D. binata* plants requires the participation of other compounds that modify the chitin substrate for degradation by the DbChitI-3 enzyme. Expansins, which effectively loosen polysaccharide networks under low pH conditions including chitin, might play a role. Expansins of both plant (Marowa et al. 2016) and microbial origin (Tovar-Herrera et al. 2015) have been identified to remove numerous inter- and intramolecular hydrogen bonds between linear polysaccharide chains. In carnivorous plants, they may synergistically aid chitinases in decomposing chitin substrates to effectively capture insect prey. In non-treated *D. binata* plants, *DbChitI-3* expression was detected in leaf blades, low in petioles, and was almost absent in roots and inflorescences. The fact that the expression of *DbChitI-3* is restricted to the leaf blades densely populated with tentacles (digestive organs) supports its specialization in digestive physiology.A similar expression profile was also detected in other sundew species, *D. rotundifolia* (Jopcik et al. 2017) and *D. adelae* (Arai et al. 2021), with the highest amount of chitinase class I mRNA transcripts found in the tentacles. Organ-specific expression of the *DbChitI-3* gene can be regulated using one of three models proposed by Nishimura et al. (2013) based on a study of the spatial expression profile of S-like *RNase* and its orthologs in *D. adelae*, *Cephalotus follicularis*, and *Dionaea muscipula*. The proposed models involve (1) constitutive expression in tentacles due to the promoter sequence demethylation selectively in the trap organ, (2) constitutive expression in tentacles due to the permanent presence of the activator and (3) activator-induced expression in traps. The expression profiles of chitinase class I *DbChitI-3* and *DrChit,* in *D. binata* and *D. rotundifolia*, respectively, correspond most closely to the third model, although slight differences were observed in their activation by applied inducers.

Unlike *DrChit* in *D. rotundifolia*, where chitin was a stronger inducer of gene transcription than gelatin (Jopcik et al. 2017), differences in the mRNA transcript levels of *DbChitI-3* in *D. binata* were not statistically significant after treatment with the specific and non-specific inducers (chitin vs gelatin and pachyman); all were potent inducers of *DbChitI-3* transcriptional activity in leaves of *D. binata.* When class III and IV chitinases in plants of the genus *Nepenthes* were tested, chitin acted as a weak or no inducer of their expression activity (Saganova et al. 2018). This might be because the class I chitinases show relatively high enzyme activity towards chitin substrates (Rajninec et al. 2020), whereas chitinases of classes III and IV show only weak activity towards polymeric substrates (Ishisaki et al. 2012a, b). Mechanic inducer – sand exhibited a different effect on the *DbChitI-3* expression than the *DrChit* gene, although both encode class I chitinase with similar molecular mass but different isoelectric points. It suggests that chitinase's basic/acidic character might be associated with a specific role and corresponding gene regulation during digestion.

Surprisingly, the non-specific substrate pachyman (β−1,3-glucan) induced a higher abundance of *DbChitI-3* mRNA transcripts than the specific substrate chitin, with a peak at 24 h post-application. Similarly, a non-specific inducer was found to be functional for chitinases of the III and IV classes in *Nepenthes* plants when their strong up-regulation was due to the presence of proteins or ammonium (Saganova et al. 2018). The β−1,3-glucans are standard terrestrial and aquatic biomass components with various biological functions. They can be distinguished by the degree of their polymerization and branching, while they are components of the cell wall of bacteria (curdlan), algae (laminarin), euglens (paramylon), and fungi (pachyman), but not of the exoskeleton of insects. The results of the experiments with pachyman and laminarin showed that only the structurally more complex pachyman [DP ~ 255, (Saito et al. 1968)] than laminarin [DP ~ 20 – 30, (Nelson and Lewis 1974)] led to the induction of *DbChitI-3* gene transcription. This form of β−1,3-glucan is present in the fungal cell wall and, similar to fungal pathogens, can enhance the plant immune response and induce the expression of pathogenesis-related proteins, including chitinases (Shetty et al. 2009; Chavanke et al. 2022). The expression response of the *DbChitI-3* gene to the presence of the pachyman supports molecular evolutionary studies that some of the genes initially involved in defence against fungal pathogens in the ancestors of carnivorous plants have duplicated and diverged during the evolution of carnivory (Renner and Specht 2012; Pavlovic and Mithoefer 2019). Moreover, a striking similarity between plant defence and carnivory has also been found in signal transduction pathways. In plants, resistance to biotrophic and necrotrophic pathogens is controlled by the salicylic and jasmonic acid pathways, respectively (Kunkel and Brooks 2002; Dietrich et al. 2004). Within the order Caryophyllales, in the case of carnivory, the plants harness the jasmonic acid-signalling pathway to induce digestive enzymes (Pavlovic and Saganova 2015; Krausko et al. 2017; Kocáb et al. 2021). Nevertheless, both these hormones have been identified as a strong up-regulators of genes for numerous groups of WRKY transcription factors with a binding preference for the WBOX (C/T)TGAC(C/T) in promoter sequences (Eulgem et al. 2000; Jiang et al. 2017; Liu et al. 2017). Scanning the *DbChitI-3* promoter sequence revealed nine W/WRKY boxes within the ~ 300 nucleotide sequence upstream of TSS (Table S2). Similarly, a frequent occurrence of W/WRKY boxes was detected in the promoter sequence of *D. rotundifolia* chitinase, which also plays a role in carnivory (Jopcik et al. 2017).

Laminarin (β−1,3-glucan with DP ~ 20—30) was detected as an efficient trigger of defence mechanisms in several non-carnivorous plant species, including tobacco (Klarzynski et al. 2000), rice (Inui et al. 1997) and tea (Xin et al. 2019). In carnivorous *D. binata* plants, laminarin, unlike pachyman did not induce the expression of the digestion-associated extracellular chitinase class I (*DbChitI-3*). It has been suggested that available β−1,3-glucanases can more easily hydrolyze the substrate with a lower DP (Michalko et al. 2013) and that hydrolysis products can negatively affect the transcription of genes involved in digestion.

This notion was confirmed in our experiments with the monosaccharide D( +)-glucose when its application at concentrations of 10 mM and 100 mM resulted in a different transcriptional activity of the *DbChitI-3* gene, while a higher amount of glucose decreased its gene transcription (Fig. 7c, Fig. S2). The same inhibitory effect was shown by 100 mM N-acetyl-D-glucosamine and 2% laminarin (w/v) (Fig. 7d,e). Finally, the experiment with a mixture of inducer and suppressor [pachyman and D( +)-glucose] of the *DbChitI-3* gene revealed that absorption of monosaccharide nutrients in leaves of *Drosera* plants is preferred and even temporarily leads to the suppression of the expression of digestion involved chitinase. We can speculate that the entire cluster of prey-induced genes may have a similar expression profile, where up- and down-regulation are controlled by presence/absence of digestion inducers and monosaccharide nutrients, respectively.

In conclusion, this study showed that *DbChitI-3* gene encoding class I acid chitinase exhibits its gene activity in leaves with digestive organs, tentacles. Within chitinase enzymes class I involved in the digestive processes of *D. rotundifolia* and *D. binata*, differences exist in enzyme characteristics and substrate specificity. While the basic chitinase of *D. rotundifolia* cleaves chitin powder at pH 6.0, the acidic chitinase from *D. binata* effectively cleaves acetic acid-swollen chitin at low pH. Specific expansins likely support the enzymatic hydrolysis of chitin biomass during digestive processes in *D. binata* plants, particularly at low pH. Treatment of leaves by specific substrate chitin or some non-specific compounds (gelatin, pachyman) mimicked the presence of insect prey and resulted in up-regulation of the *DbChitI-3* gene compared to the control, while the maximum of mRNA transcripts was detected 24 h after inducer application. In contrast, treating leaves with laminarin and monosaccharides decreased *DbChitI-3* gene transcription compared to the control. This indicates that the regulation of digestion is a complex process with different inducers and suppressors, while the products of the enzymatic reactions of hydrolases are among the most determining components of fine-tuning. Chitin recognition is conserved in plants, while several lysine-motif-containing receptors have been identified in the activation of plant cell response (Miya et al. 2007). The mechanisms underlying β-glucan recognition remain largely unexplored as they depend on its origin and plant species (Wanke et al. 2020). Identifying specific receptors that recognize β−1,3-glucan structures in potential prey is still challenging.

## Supplementary Information

Below is the link to the electronic supplementary material.

Supplementary file1 (DOCX 62 KB)

## Data Availability

All data generated or analyzed during this study are provided in this published article and its supplementary data files.

## Abbreviations

DbChitI-3: Drosera binata chitinase class I
FITC-chitin: N-fluorescein-labelled chitin
4-MUF-NAG: 4-Methylumbelliferyl N-acetyl-β-D-glucosaminide
4MU-(GlcNAC)_2_: 4-Methylumbelliferyl N,N′-dicetylchitobioside
4MU-(GlcNAC)_3_: 4-Methylumbelliferyl β-D-N,N′,N″-triacetylchitotrioside
