# Supplementary material for: Complex transcription regulation of acidic chitinase suggests fine-tuning of digestive processes in Drosera binata
Source: Planta. 2025 Jan 12;261(2):32. doi: 10.1007/s00425-025-04607-2 (PMC11725546; doi:10.1007/s00425-025-04607-2)
Supplement: Supplementary file 1 — Supplementary file1 (DOCX 62 KB) [file 425_2025_4607_MOESM1_ESM.docx]

**Supporting Information**

**Table S1.** Primers used for the isolation of *DbChitI-3* (full-length, cDNA, cds) and expression analysis.

| **Primer** | **Sequence (5´­ 3')** | **Expected size** | **Purpose** |
| --- | --- | --- | --- |
| **P1FOR** | TAGTGGAGGTGGTGATGTCAGCT | 868 bp | Amplification of genomic region of *DbChitI-3* gene |
| **P2REV** | Tcagcagcagaaggagtccattgt |  |  |
| **AP1FOR/REV** | GTAATACGACTCACTATAGGGC | 379 bp  (*EcoR*V library) | Overlapping 5' *DbChitI-3* sequence |
| **AP2FOR/REV** | ACTATAGGGCACGCGTGGT |  |  |
| **P3REV** | AAATCCAGGGAAAGACCTTGCGGCTtgaat |  |  |
| **P4REV** | aggacaagcattgtcgtttcgatgaagaa |  |  |
| **P5REV** | ACTGCTCCGCCAGCCTGATAGCCACATTGT | 385 bp  (*Dra*I library)  1377 bp  (*Stu*I library) | Overlapping 5' regulatory sequence of *gDbChitI-3* |
| **P6REV** | TAGCAGGATACAAATCCTCATGGTTATGAT |  |  |
| **P7REV** | ATTTGTGGCGCAAAGTTGGCAGAAAT |  |  |
| **P8REV** | TTCTCACCCTATGTTTAGGTGGAAACTT |  |  |
| **P9FOR** | tacaactacggacaatgtggcgctgccat | 433 bp  (EcoRV) | Overlapping 3' regulatory sequence of *gDbChitI-3* |
| **P10FOR** | AACCATTGTTGGCGAACCCGGATTTGGTA |  |  |
| **P11FOR** | ATATTAGACTGAGTCCTTACTGAT | 2938 bp | Integrity verification of *gDbChitI-3* |
| **P12REV** | TTGGAGAGTTCCAATGCGTATCA |  |  |
| **P13FOR** | cgactggagcacgaggacact | 283 bp | Identification of 5´-transcription start of the *DbChitI-3* gene |
| **P14REV** | TTCTTTCGTGTGTTTATGTCACCCGTGGT |  |  |
| **P15FOR** | ggacactgacatggactgaaggagta |  |  |
| **P16REV** | TGCAACACAGCCCATTTGGGCAAACTGCT |  |  |
| **P17FOR** | TCAAGGAAGAACAAGGCAATCCAGGTTCTT | 248 bp | Identification of 3´-transcription stop of the *DbChitI-3* gene |
| **P18REV** | gctgtcaacgatacgctacgtaacg |  |  |
| **P19FOR** | AACAAACCGTCGTGCCATGCTGTCATC |  |  |
| **P20REV** | cgctacgtaacggcatgacagtg |  |  |
| **P21FOR** | ACC**ATG**AGGATTTGTATCCTGCTACTGCTT | 966 bp | Isolation of *cdsDbChitI-3* gene |
| **P22REV** | TTGCATTAAGAAAAAGGACGCTGATTATTGCAA |  |  |
| **P23FOR** | TATTGCGGACCGGATGAGCAAG | 308 bp | RT-qPCR: internal control amplification of the *β-actin* cDNA fragment |
| **P24REV** | CTGACGACCCGCACCTTTCTACAC |  |  |
| **P25FOR** | TAGCCTCTAGCACTTACCCTTGCGC | 259 bp | RT-qPCR: amplification of the *DbChitI-3* cDNA fragment |
| **P26REV** | GCTTGGTCAGCAGAAGAAGGACTCC |  |  |
| **P27FOR** | aacacgtgGAACAATGTGGCTATCAGGCTGGCGG | 915 bp | Amplification of *DbChitI-3* cDNA fragment for expression in *E. coli* |
| **P28REV** | aacacgtgAGGACGCTGATTATTGCAATCTAGGTT |  |  |
| **P29FOR** | CTGTCAGACCAAGTTTACTCAT | 4481 bp | pET-K1 vector preparation |
| **P30REV** | CACATTTCCCCGAAAAGTGCC |  |  |
| **P31FOR** | GTACTAAAACAATTCATCCA | 825 bp |  |
| **P32REV** | CTGTAGAAAAGAGGAAGGA |  |  |
| **P33FOR** | CACCACCACCACCACCACTGAGA | 5251 bp | pET-K2 vector preparation |
| **P34REV** | CACGTGGTGATGGTGATGGTGATGCATATG |  |  |

*underlined are sequences for the *Pml*I restriction enzyme

|  |  |
| --- | --- |

**Table S2** The *cis*-regulatory elements with putative functions identified within the both strands (+), (-) of the 1022-bp *DbChitI-3* promoter sequence predicted using the PLACE database. The position of the TIS is +1 and the nucleotides upstream from this site are negatively numbered.

| ***Cis*-element** | **Location** | **Signal sequence** | **Putative function** |
| --- | --- | --- | --- |
| CAAT | +18 (+), +137(+), -153 (+), -285 (+), -826 (+), -861 (+), +60 (-), -7 (-), -214 (-), -243 (-), -411 (-),-440 (-) -687 (-), -713 (-), | CAAT | Common *cis*-acting element in promoter and enhancer regions |
| GATABOX | -228 (+), -263 (+),-875 (+), -902 (+), -369 (-), -667 (-) | GATA | Light-responsive elements |
| TBOX | -53 (+), - 537 (+) | ACTTTG |  |
| CIACADIANLELHC | -15 (-), -222 (-), -709 (-) | CAANNNNATC |  |
| GT1 | -68 (+),-262 (-), -595 (+),639 (+), -700 (+), -724 (+), -874 (+), -981 (+), -188 (-), -239 (-), -300 (-), -307 (-), -659 (-), -917 (-)- | GRWAAW |  |
| RAV1AAT | -324(+) | CAACA | Root-specific element |
| ROOTMOTIF | -65 (+), -331 (+),-445 (+),-476 (+),-260 (-) | ATATT |  |
| POLLEN1LELAT52 | -80 (+), -418 (+), -740 (+), -62 (-), -290(-), -299(-), -907(-) | AGAAA | Pollen-specific elements |
| GTGANTG10 | -238 (+), -360 (+), -422 (+), -435 (+), 481 (+), -176 (-), -452 (-) | GTGA |  |
| ARR1AT | -16 (+), -218 (+), -223 (+), -680 (+), 717 (+), 433 (-), -641 (-), -871 (-),-979 (-) | NGATT | Cytokinin-responsive element |
|  |  |  |  |
| EBOX | -32 (+), -173 (+), -339 (+), -509 (+), -706 (+), -835 (+) | CANNTG | Dehydration-, cold- and JA- responsive element |
| MYBCORE | -75 (-), -140 (-), -350 (-), -702 (-), -726 (-), -931 (-) | CNGTTR | Dehydration responsive element |
| ACGTATERD1 | -462 (+), -953 (+) | ACGT | Dehydration early responsive element |
|  |  |  |  |
| WBOX | -8 (+), -9 (+), -162 (+),-805 (+), -809 (+), -886 (+), -887 (+)-863 (-), ´-870 (-) | T)TGAC(Y) | disease-responsive element |

**Legend:** W = A or T; Y = C or T; R = A or G; K = G or T

(C/T)TGAC(C/T)

**Fig. S1** Amino acid sequences of the *D. binata* chitinase gene open reading frame (DbChitI-3), mature protein without signal peptide (DbChitI-3^S-^), and recombinant protein with His tag (rDbChitI-3^S-^His).

>DbChitI-3

MRICILLLLCVSLFLSGALAEQCGYQAGGAVCPNGLCCSQYGYCGTTSAYCGSGCQSQCGGGSPPPSPPLPPSPSGGGDVSSIITSQIFNEMLLHRNDNACPANGFYTYDAFIQAARSFPGFGTTGDINTRKKELAAFFGQTSHETTGGWPTAPDGPYAWGYCFKEEQGNPGSYCVASSTYPCAPGKKYYGRGPIQISYNYNYGQCGAAINQPLLANPDLVASNADISFETAIWFWMTPQGNKPSCHAVITGQWSPSSADQAAGRVPGYGVITNIINGGIECGKGQNAEAANRIGFYERYCSLLGISPGNNLDCNNQRPFS-

**>**DbChitI-3^S-^

EQCGYQAGGAVCPNGLCCSQYGYCGTTSAYCGSGCQSQCGGGSPPPSPPLPPSPSGGGDVSSIITSQIFNEMLLHRNDNACPANGFYTYDAFIQAARSFPGFGTTGDINTRKKELAAFFGQTSHETTGGWPTAPDGPYAWGYCFKEEQGNPGSYCVASSTYPCAPGKKYYGRGPIQISYNYNYGQCGAAINQPLLANPDLVASNADISFETAIWFWMTPQGNKPSCHAVITGQWSPSSADQAAGRVPGYGVITNIINGGIECGKGQNAEAANRIGFYERYCSLLGISPGNNLDCNNQRPFS-

>rDbChitI-3^S-^His

HHHHHHHVEQCGYQAGGAVCPNGLCCSQYGYCGTTSAYCGSGCQSQCGGGSPPPSPPLPPSPSGGGDVSSIITSQIFNEMLLHRNDNACPANGFYTYDAFIQAARSFPGFGTTGDINTRKKELAAFFGQTSHETTGGWPTAPDGPYAWGYCFKEEQGNPGSYCVASSTYPCAPGKKYYGRGPIQISYNYNYGQCGAAINQPLLANPDLVASNADISFETAIWFWMTPQGNKPSCHAVITGQWSPSSADQAAGRVPGYGVITNIINGGIECGKGQNAEAANRIGFYERYCSLLGISPGNNLDCNNQRPFSHVHHHHHH


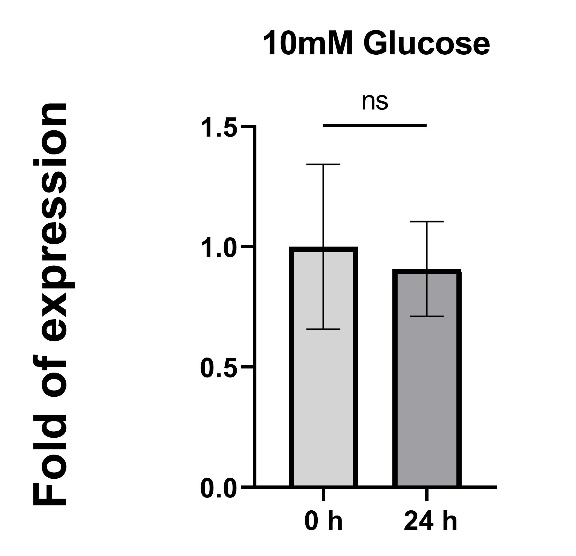


**Fig. S2** The *DbChitI-3* mRNA transcription level in the leaves of *Drosera binata* upon application of 10 mM glucose after 24 h. The basal expression refers to non-treated control leaves, referred to as 1. Data from RT-qPCR were normalized relative to the abundance of the endogenous control gene β-actin. Error bars depict standard errors (*n* = 4). The difference was insignificant at * *P* ˂ 0.05 compared to the control (untreated leaf blades).
